# Establishment of clinical pharmacy services: evidence-based information from stakeholders

**DOI:** 10.1186/s12960-023-00887-5

**Published:** 2024-01-10

**Authors:** Manase Kilonzi, Ritah F. Mutagonda, Dorkasi L. Mwakawanga, Hamu J. Mlyuka, Wigilya P. Mikomangwa, Wema A. Kibanga, Alphonce Ignace Marealle, Bertha Mallya, Deogratias Katabalo, Sofia Sanga, Fredrick Kalokola, John Rwegasha, Rose Magambo, John Mmassy, Sungwa Kabissi, Josephine A. Balati, Peter Maduki, Omary Mashiku Minzi, Appolinary A. R. Kamuhabwa

**Affiliations:** 1 School of Pharmacy, Muhimbili University of Health and Allied Sciences, P. O. BOX 65013, Dar es Salaam, Tanzania; 2 School of Nursing, Muhimbili University of Health and Allied Sciences, P. O. BOX 65001, Dar es Salaam, Tanzania; 3 School of Pharmacy, Catholic University of Health and Allied Sciences, P. O. BOX 1464, Mwanza, Tanzania; 4 Department of Internal Medicine, Muhimbili National Hospital, P. O. BOX 65000, Dar es Salaam, Tanzania; 5 School of Medicine, the Catholic University of Health and Allied Sciences, P. O. BOX 1464, Mwanza, Tanzania; 6 Christian Social Services Commission, P.O. BOX 9433, Dar es Salaam, Tanzania

## Abstract

High morbidity and mortality related to the use of drugs resulted in demand for clinical pharmacy services (CPS) globally. In developed countries, the evolution of pharmacists’ role in direct patient care started in the 1960s. The participation of pharmacists in CPS has resulted in positive clinical, economic, and humanistic outcomes. In developing countries, efforts have started to ensure pharmacists are engaged in the provision of CPS. However, the efforts are hampered by poorly defined pharmacist career paths, financial constraints, and a lack of political willingness. In Tanzania, efforts started in 2008, in which CPS was introduced into the Bachelor of Pharmacy curriculum, followed by the initiation of a postgraduate program on hospital and clinical pharmacy in 2013. A regulation was released by the Tanzania Ministry of Health in 2020 to enforce pharmacists' engagement in providing CPS. In 2021, a project was launched in the country, aiming to strengthen the provision of CPS in public and faith-based hospitals by training on-job pharmacists. The project was implemented in phases, including stakeholders’ engagement, baseline survey, training, and supportive supervision of the trained pharmacists. Therefore, this commentary aims to share what we experienced during project implementation, the achievements, challenges, and key lessons learned.

## Global overview of clinical pharmacy services

Clinical pharmacy service (CPS) is offered by a pharmacist when deciding and monitoring a patient’s pharmacotherapy [1]. Globally, CPS started after a surge increase in the burden of drug-related morbidity and mortality. Among countries that accepted and integrated the services earlier are the United States of America, Canada, Spain, and Brazil [1]. Later, CPS was adopted in Europe, Asia, and African countries like South Africa in the early 1990s [2]. CPS requires pharmacists to optimize patients' medication therapy and promote health, wellness, and disease prevention [3]. The major areas where CPS is well established globally are hospitals, ambulatory care facilities, and community pharmacies [1].

In developed countries where CPS is well established, a reduction of adverse drug reactions (ADRs), medication errors, hospital stay, hospital readmission, and drug–drug, and drug–food interactions have been evidenced [4]. Besides, CPS promotes medication adherence, lowers treatment costs, fights emergency antibiotic resistance, builds the reputation of the facilities, and raises public confidence in the healthcare system [5]. Although CPS is adequately implemented in developed countries, literature reports the existence of some barriers to be addressed. These include inadequate pharmacist training, poor mentorship and peer support, pharmacist personality, lack of pharmacist-defined roles, mistrust among healthcare providers (HCPs), and shortage of resources and funds [6].

In low-middle-income countries (LMICs), the role of pharmacists is also evolving from ordinary manufacturing, supply chain, compounding, and dispensing to the provision of CPS [7]. Although LMICs have adopted the concept of CPS, its implementation faces several challenges: lack of guiding policies, universities' use of old curricula, shortage of staff, inadequate motivation scheme, and lack of on-the-job training [8]. Other barriers mentioned by the literature are inadequate clinical and communication skills, assertiveness, negative attitudes and perceptions among pharmacists, and the historical dominance of medical doctors in healthcare systems [6, 8, 9].

## Status of CPS provision in Tanzania

In Tanzania, the provision of CPS is at the infant stage. In 2008, the Muhimbili University of Health and Allied Sciences (MUHAS), a premier medical university in Tanzania, revised its Bachelor of Pharmacy curriculum to include the components of CPS. Five years later, MUHAS established a Master of Pharmacy Degree in Hospital and Clinical Pharmacy. However, the program faces the challenge of low enrollment of pharmacists. To support the effort to increase the number of clinical pharmacists in Tanzania, the Ministry of Health initiated a sponsorship program for government-employed pharmacists who enroll in the Hospital and Clinical Pharmacy Masters program. However, pharmacists are not motivated to join the program, and among the drawbacks is a lack of remuneration and recognition as specialists in pharmacy in the scheme of services.

In 2020, the Ministry of Health in Tanzania, through the Pharmacy Council, amended the pharmacy regulations to include CPS as one of the core functions of pharmacists. The regulations outlined pharmacists’ general duties and responsibilities, including participation in therapeutic management, multidisciplinary ward rounds, pharmacist-only ward rounds, and detection and reporting of ADRs [10]. Despite all the efforts mentioned above, the provision of CPS in Tanzania remains low.

In 2021, MUHAS, in collaboration with the Christian Social Services Commission (CSSC) and with the support of action medeor e.V of Germany, implemented a project to strengthen the provision of CPS in public and faith-based hospitals in Tanzania. Therefore, the purpose of this commentary is to share steps taken to engage pharmacists in the provision of CPS during the project, the achievements, challenges, and critical lessons learned. The information will serve as a reference to other LMICs struggling to engage pharmacists in the provision of CPS.

## Project overview

The project was implemented from August 2021 to December 2022. The implementation process had 5 phases: engagement of stakeholders, baseline survey, development of CPS short course curriculum for on-the-job pharmacists, training of the pharmacists, and supportive supervision of the trained pharmacists. Key stakeholders influencing the engagement of pharmacists in CPS provision, either directly or indirectly, were identified and informed of the project. Input from the stakeholders’ discussion was used to strengthen tools for the baseline survey. Then, a baseline survey was carried out to identify the level of implementation of CPS in public and faith-based hospitals. The survey assessed the level of knowledge, attitude, and practice (KAP) of pharmacists in the provision of CPS. It also explored barriers, facilitators, and strategies to improve the provision of CPS in Tanzania.

Through the baseline survey, barriers hindering the provision of CPS were uncovered and categorized into two groups, which were individual and organizational barriers. Individual barriers encompass limited skills and lack of confidence among pharmacists, negative attitudes towards CPS, poor communication, and issues related to inferiority and superiority complexes among HCPs. Organizational barriers include the absence of on-the-job training and motivation, while external factors involve a shortage of pharmacists and the absence of CPS guidelines and standard operating procedures [6]. Nevertheless, the study highlights some facilitators to enhance CPS provision, which includes the readiness of hospital administrations to support CPS, the acceptance of CPS by nurses and doctors, and recognition of the CPS by the Tanzania Pharmacy Act and regulations [6].

The findings of the survey were presented in a stakeholders' meeting in October 2021. The meeting was chaired by the Minister of Health in Tanzania. As an immediate intervention to enhance the provision of CPS in Tanzania, stakeholders recommended the development of a curriculum for a short course in CPS training for on-the-job pharmacists in the hospitals. A 2-week course curriculum was developed and accredited by MUHAS in December 2021. The curriculum was subsequently endorsed by the Pharmacy Council of Tanzania. Before training, pharmacists, senior pharmacists, and physicians from the five zonal referral hospitals in Tanzania's mainland were identified and selected as trainers. The 11 identified trainers participated in a one-week training of trainers (ToTs) to become acquainted with the curriculum, training materials, and tools for assessment.

On-the-job training of pharmacists for the provision of CPS started in January to July 2022. A total of 104 pharmacists working in 26 public regional, zonal, and national referral hospitals were trained. The training took place in zonal and national referral hospitals where the ToTs were selected. Pharmacists working in regional referral hospitals were invited to participate in the training at the respective zonal or national hospitals.

Before the beginning of training, the head of the facility (medical officer in charge/director) and the team comprising the clinical services coordinator and the head of the nursing unit were invited to officiate and be orientated to the training. The training consisted of three days of lectures, discussions, demonstrations, and seven days of hands-on training. The hands-on training involved attending ward rounds and clinics with other HCPs of that training center. Nurses, medical doctors, and senior pharmacists from MUHAS and selected hospitals were engaged as trainers during ward rounds and clinic visits. In addition, facilitators and trainees had at least 60 min each day to receive and discuss feedback from the trainees participating in ward rounds and clinics. The discussion focused on clinical cases encountered, challenges, and mitigation strategies.

Three months after training in each hospital, supportive supervision was conducted in 17 of the 26 hospitals where pharmacists were trained. During supportive supervision in the hospitals, discussions were held with the head of the hospital, the head of the pharmacy department, and the trained pharmacists. Information was collected regarding the implementation of CPS activities at the hospitals after the training and the challenges encountered. The head of the pharmacy department and the trained pharmacists were given a checklist, which was used to determine the level of implementation of CPS in the hospital. The checklist included indicators such as the establishment of a CPS unit, the presence of a CPS focal person, the presence of a ward-round schedule for pharmacists, the presence of documented cases encountered during ward rounds, and evidence for the pharmacist’s weekly case presentation (Table 1).

**Table 1:** Supportive supervision indicators for CPS in hospitals (visited hospitals = 17)

Variables	Yes	No
The provision of CPS already started	17	0
Ward-round timetables/schedules available	Yes = 10	No = 7
Pharmacists only ward-round conducted	Yes = 0	No = 17
CPS unit established	Yes = 1	No = 16
The CPS focal person appointed	Yes = 6	No = 11
Pharmacists assigned to participate in ward rounds and clinics	Yes = 6	No = 11
Documentation of cases observed during ward rounds and visitation to the clinics	Yes = 4	No = 13

## Achievements, challenges, and key lessons learned from the project

Analysis of the supportive supervision findings revealed that 5 of the 17 visited hospitals have created a system for pharmacists to access patient information through the hospital information management system (HIMS). Three hospitals have created space in the patients` files for pharmacist’s notice, 6 have appointed CPS focal persons, and 1 has established a CPS unit (Table 1). In the hospitals where supportive supervision was conducted, nurses and medical doctors reported that pharmacists need to be involved in ward rounds and clinics. This demonstrates the importance of pharmacists' participation in optimizing patient care in the context of the shortage of HCPs. Post-training challenges that limit trained pharmacists from the provision of CPS include a shortage of pharmacists and intern pharmacists, limited skills among pharmacists who did not attend training, lack of readiness among pharmacists, lack of access to patient information through HIMS by pharmacists, and absence of space in the patient file for pharmacists to document their interventions for clinicians’ considerations**.**

In conclusion, it is possible to establish and implement CPS in LMICs. However, due to other pharmaceutical responsibilities facing pharmacists in hospitals, the engagement of pharmacists in CPS should be adequately planned and coordinated. The ministries responsible for health and higher learning education should consider CPS as one of the core functions of pharmacists and minimize the identified barriers. Nevertheless, stakeholders should join hands to ensure the adequate provision of CPS. Lastly, pharmacists in LMICs are encouraged to practice CPS despite the existing challenges.

## Data Availability

Not applicable.

## Abbreviations

ADRs: Adverse drug reactions
CPS: Clinical Pharmacy Services
CSSC: Christian Social Services Commission
HCPs: Healthcare providers
HIMS: Hospital Information Management System
LMICS: Low-middle income countries
MUHAS: Muhimbili University of Health and Allied Sciences
ToTs: Training of trainers
